# Bacterial Communities Associated with the Leaves and the Roots of *Arabidopsis thaliana*

**DOI:** 10.1371/journal.pone.0056329

**Published:** 2013-02-15

**Authors:** Natacha Bodenhausen, Matthew W. Horton, Joy Bergelson

**Affiliations:** 1 Department of Ecology and Evolution, University of Chicago, Chicago, Illinois, United States of America; U. S. Salinity Lab, United States of America

## Abstract

Diverse communities of bacteria inhabit plant leaves and roots and those bacteria play a crucial role for plant health and growth. *Arabidopsis thaliana* is an important model to study plant pathogen interactions, but little is known about its associated bacterial community under natural conditions. We used 454 pyrosequencing to characterize the bacterial communities associated with the roots and the leaves of wild *A. thaliana* collected at 4 sites; we further compared communities on the outside of the plants with communities in the endophytic compartments. We found that the most heavily sequenced bacteria in *A. thaliana* associated community are related to culturable species. *Proteobacteria*, *Actinobacteria*, and *Bacteroidetes* are the most abundant phyla in both leaf and root samples. At the genus level, sequences of *Massilia* and *Flavobacterium* are prevalent in both samples. Organ (leaf vs root) and habitat (epiphytes vs endophytes) structure the community. In the roots, richness is higher in the epiphytic communities compared to the endophytic compartment (*P* = 0.024), while the reverse is true for the leaves (*P = *0.032). Interestingly, leaf and root endophytic compartments do not differ in richness, diversity and evenness, while they differ in community composition (*P = *0.001). The results show that although the communities associated with leaves and roots share many bacterial species, the associated communities differ in structure.

## Introduction

Biotic factors such as host species [1]–[3], genotype [4]–[6], and leaf age [7], [8] can all impact the structure of microbial communities associated with plants, as can the many abiotic factors that influence the physiology of host plants (e.g. [9], [10]). One might expect the importance of particular abiotic factors to vary, depending upon the location of the microbial community within the plant, and this may have repercussions for the structure of microbial communities. For example, the microbial community residing in the phyllosphere (the aerial parts of plants) is faced with a nutrient poor and variable environment that is characterized by fluctuating temperature, humidity and UV radiation [11]. The microbial community in the rhizosphere (the soil directly in contact with the roots), on the other hand, resides within a stable environment that is rich in nutrients due to the chemicals exuded by plants to attract beneficial microorganisms and combat pathogens [12]. If environmental variability promotes diversity, as has been suggested [13], [14], then the microbial community within the phyllosphere would be predicted to be more diverse than that within the rhizosphere. There is a little evidence that touches on this hypothesis; however, there are few direct comparisons of rhizosphere and phyllosphere bacterial communities, especially comparisons using material from the same plants [15], [16].

Microbial communities colonizing plants may protect them against pathogen infection [15], [17], and *Arabidopsis thaliana* is an important model to study plant defense against pathogens. A characterization of the bacterial communities colonizing *Arabidopsis thaliana* in the field is therefore valuable, not only for exploring how ecological factors shape communities but also for its applied relevance. To date, the bacterial community of the rhizosphere soil associated with *A. thaliana* has been studied using fingerprinting methods [18], and the bacterial community of the phyllosphere has been characterized with DGGE and clone libraries [19]. However, for both studies, plants were grown in growth cabinets, and therefore, lack potential colonizers. Of more relevance to naturally occurring bacterial communities, Delmotte *et al*. [20] found that *Methylobacterium*, *Sphingomonas* and *Pseudomonas* proteins were the most abundant in the phyllosphere of *A. thaliana* grown in the field at one site. None of the three studies above compared communities on the surface (epiphytic) and within the plants (endophytic). Two studies that did compare bacterial communities associated with the inside and the outside of *A. thaliana* roots used 454 pyrosequencing to find that the endophytic compartment harbors a less diverse community than the rhizosphere [21], [22]. In this study, we also characterized bacterial communities using 454 pyrosequencing of the 16S rRNA gene. We obtained more than 4000 sequences per sample. Our main objective was to describe and compare epiphytic and endopyhtic bacterial communities associated with the roots and leaves of *A. thaliana* growing under natural conditions.

## Materials and Methods

### Site Description and Sampling

*Arabidopsis thaliana* samples were collected at 4 sites: on April 15 2008 at Route Marker 166 (41°21'17.41"N, 86°44'14.47"W), on April 16 2008 at Lake Michigan College (42° 5'24.41"N, 86°23'36.27"W), on April 25 2008 at Michigan Extension (42°5'33.72"N, 86°21'22.76"W), and on April 30 2008 at North Liberty (41°32'24.88"N, 86°25'32.86"W). All of these sites are disturbed. The described field study did not require specific permits and did not involve endangered or protected species. The locations are not privately-owned or protected in any way. In the Midwest, *A. thaliana* germinates at the end of the summer and overwinters as a rosette. Samples were harvested a few weeks after the snow melt. The plants were healthy-looking, although reddish, indicating that the plants were stressed, potentially from the cold. They were also smaller than *A. thaliana* grown in growth chambers, which is typical of field collected *A. thaliana*. Root sizes varied, with plants from Root Marker 166 having the largest roots.

At each site, we collected approximately 20 plants with sterile gloves. The roots were cut from the rosettes in the field using a sterile razor blade and both sample types were stored in sterile 50 ml polystyrene tubes. Plants from one site were bulked into one sample. Samples were brought to the lab on ice, and then stored at −80°C before processing.

Microbes living on the plant surface (epiphytes) were separated from microbes living within the plant (endophytes) using a modified protocol [23]. For the rosette samples: samples were weighed and for each gram of leaf material, 10 ml of 0.1 M potassium phosphate buffer, pH 8.0 was added to the tubes. Tubes were sonicated 1 min and vortexed for 10 sec; this procedure was repeated twice. The wash steps were repeated once. The leaf wash was then filtered using a 115-ml (0.2 um) nitrocellulose filter unit (Nalgene, NY, USA). The roots were washed in a similar way, except the samples (roots and the most tightly associated sand, comprising <1 mm of sand surrounding the roots) were first placed in the filter unit and washed twice with 10 ml phosphate buffer. Roots were then removed from the filter unit and placed in 50-ml vials with 20 ml phosphate buffer, sonicated, and vortexed 6 times every 5 minutes. The root wash was then filtered using the filter unit. Root and leaves were washed twice with 70% ethanol, then stored at −80°C for later extraction of the endophytic fraction.

### DNA Extraction, PCR Amplification, and Sample Pooling

Altogether, there are 16 DNA samples: plants were collected from 4 sites, for each site, there are both root and leaves, and for each organ, there are epiphytic and endophytic fractions. For the epiphytic fraction, DNA was extracted from half of the filter using the Power Soil DNA kit (MoBio Laboratories). For the endophytic fraction, each sample was pulverized in liquid nitrogen with a mortar and pestle. An aliquot (150 mg) of each sample was added to the bead tubes from the Power Soil DNA kit (MoBio Laboratories), followed by extraction with the standard MoBio protocol. DNA concentration was determined using PicoGreen (Invitrogen). DNA concentration was adjusted to 10 ng/µl for the endophytic samples and 1 ng/µl for the epiphytic samples; a lower concentration is used for the epiphytic samples because the DNA is mostly microbial DNA. The DNA of the endophytic samples on the other hand also includes plant DNA.

Primer 799F (5'-AACMGGATTAGATACCCKG-3'), which minimizes contamination from plastid DNA [24] and a primer designed for this study, 1193r (5'-ACGTCATCCCCACCTTCC-3'), were used to amplify V5, V6 and V7 of the 16S rRNA gene. The forward primer was fused to the 454 Life Sciences primer B and the reverse primer was fused to the adapter A and a barcode in order to sequence the hypervariable regions V6 and V7.

Each 25 µl PCR reaction contained 10 ng (for the endophytic fraction) or 1 ng (for the epiphytic fraction) of DNA, Mg2+ free PCR buffer (TaKaRa), 3 mM MgCl2 (TaKaRa), 200 µM dNTP, 200 nM forward primer, 200 nM reverse primer, 12.5 µg ultrapure BSA (Ambion), and 1 unit Ex Taq HotStart polymerase (TaKaRa). Cycling conditions were 94°C for 2 min, followed by 25 cycles of 94°C for 30 sec, 55°C for 30 sec, 72°C for 1 min, with a final extension of 72°C for 10 min. All samples were amplified in quadruplicates, which were combined before purification. Primer 799f and 1193r amplify a mitochondrial product of about 800 bp and a bacterial product of about 500 bp. We isolated the bacterial product by separating the PCR products on a 3% low melt agarose gel (2% agarose for root samples) and excising a band of agarose with size 400 bp to 700 bp. DNA was extracted from the gel using the QIAquick gel extraction kit (Qiagen). After purification, DNA was quantified using the PicoGreen assay (Invitrogen) and the quality was checked using a Bioanalyzer (Agilent). DNA concentration was adjusted to 1 ng/µl. The amplicon libraries were prepared by pooling 10 ng of each PCR.

The amplicon libraries were sent to the High-throughput Genome Analysis Core at Joint Institute for Genomics & Systems Biology (University of Chicago/Argonne National Laboratory) for pyrosequencing on a 454 Life Sciences FL (Roche) machine. One region of the 454 run was used for DNA from the rosette samples and the other region was used for the root samples. The sequencing data have been deposited in the NCBI Sequence Read Archive (SRP018030).

### Sequence Analysis

The software package mothur (version 1.27.0) was used for sequence analysis [25] while following the Standard Operating Procedure outlined on http://www.mothur.org/wiki/Schloss_SOP. Briefly, sequencing error was reduced using shhh.flows (mothur implementation of the AmpliconNoise algorithm). Then, each unique sequence was aligned with align.seqs using the SILVA reference alignment. A distance matrix was calculated with default parameters. Chimeric sequences were identified using chimera.uchime and removed. Sequences matching “Cyanobacteria_Chloroplast" and "Mitochondria” were also removed. Next, sequences were clustered using the furthest neighbor clustering algorithm to build OTUs (operational taxonomic unit). The resulting file was parsed to separate the data for each sample. OTUs were assigned a taxonomic group with classify.seqs using the RDP reference file and a cutoff of 80% of the bootstrap value. For the description of the community, OTUs with the same taxonomy were binned together at the phylum, class and genus level.

### Statistical Analysis

Abundance tables were analyzed using the package vegan [26] within the R statistical environment (R Development Core Team; http://www.R-project.org). To test the hypothesis that none of the taxa co-occur more often than by chance [27], we transformed the species matrix using the Hellinger-transformation [28] and then calculated Kendall’s Coefficient of Concordance on the 50 most heavily sequenced taxa in each community. To estimate diversity, we minimized the impact of sequencing artifacts by restricting our analyses to all OTUs present in at least 2 samples. Percentages of sequences belonging to singletons were arcsine square root transformed before calculating the Student’s *t-test*. ANOVA was used to test the effect of ‘habitat’ (epiphyte vs. endophyte) and ‘organ’ (root vs. leaf) on the relative abundance of the members of the core community. Correspondence analysis was performed with the function *cca*. To calculate diversity indices while controlling for sampling effort, 2000 sequences were subsampled 500 times for each biological sample. For each subsampling of 2000 sequences, three diversity indices were calculated and plotted: richness, diversity and evenness. Richness (S) is the number of OTUs. Shannon-Weaver index is H = -sum pi *ln pi, where pi is the proportional abundance of species i. From the Shannon-Weaver index, one can calculate diversity: D = exp(H) [29]. Evenness was calculated with Sheldon’s evenness: E = exp(H)/S) [30]. ANOVA was used to test the effect of ‘habitat’ (epiphyte vs. endophyte) and ‘organ’ (root vs. leaf) on richness, diversity and evenness using the mean of the 500 permutations for each sample. Normality of the standardized residuals was investigated with a qqplot; furthermore, the Shapiro-Wilk test confirmed that the standardized residuals were normally distributed. Paired student’s *t-tests* were calculated for all pairwise comparisons, *P* values were adjusted using the fdr correction for multiple testing. For analysis of community composition, pairwise dissimilarities between samples were calculated based on the Bray–Curtis index with the vegan function *vegdist.* To assess the effect of ‘habitat’ and ‘organ’ on community composition, we used the vegan functions *adonis*, which is a non-parametric multivariate analysis of variance [31] as well as the functions *mrpp* [32] and *anosim* [33].

## Results

### Analysis of Pyrosequencing Data

We used the standard operating procedure from the software package mothur that includes a denoising step [25]. After removing chimeras, we obtained 135,540 sequences. We found that primer 799f amplifies both bacterial and plant chloroplast DNA under our PCR conditions; the proportion of reads assigned to a plant taxonomic identification ranges from 0 to 23% for each sample. After removing reads assigned to the taxonomic Kingdom *Plantae*, 129,445 sequences remained.

**Figure 1:**
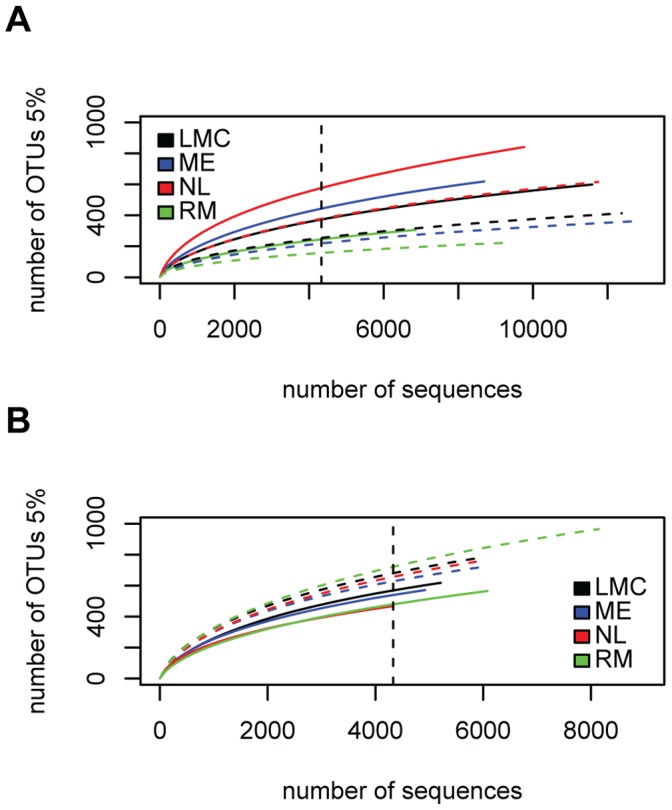
Rarefaction curves at the 5% distance cutoff. (A) Leaf-associated communities; (B) Root-associated communities. Samples were collected at 4 sites (RM, Route Marker; NL, North Liberty; ME, Michigan Extension; LMC, Lake Michigan College). Continuous lines represent endophytic samples and dashed lines represent epiphytic samples. The dashed vertical line indicates the numbers of sequences subsampled from each sample (4329 sequences).

Sequences were clustered into operational taxonomic units (OTUs) at the 0.05 distance cutoff, which is typically the genus level [34]. Rarefaction curves were starting to level off, suggesting that the plant associated communities were reasonably well characterized with our sampling effort (Figure 1). Interestingly, the rarefaction curves of the epiphytic samples are higher than the endophytic samples for the root samples while the reverse is true for the leaf samples.

### Description of the Community

**Figure 2:**
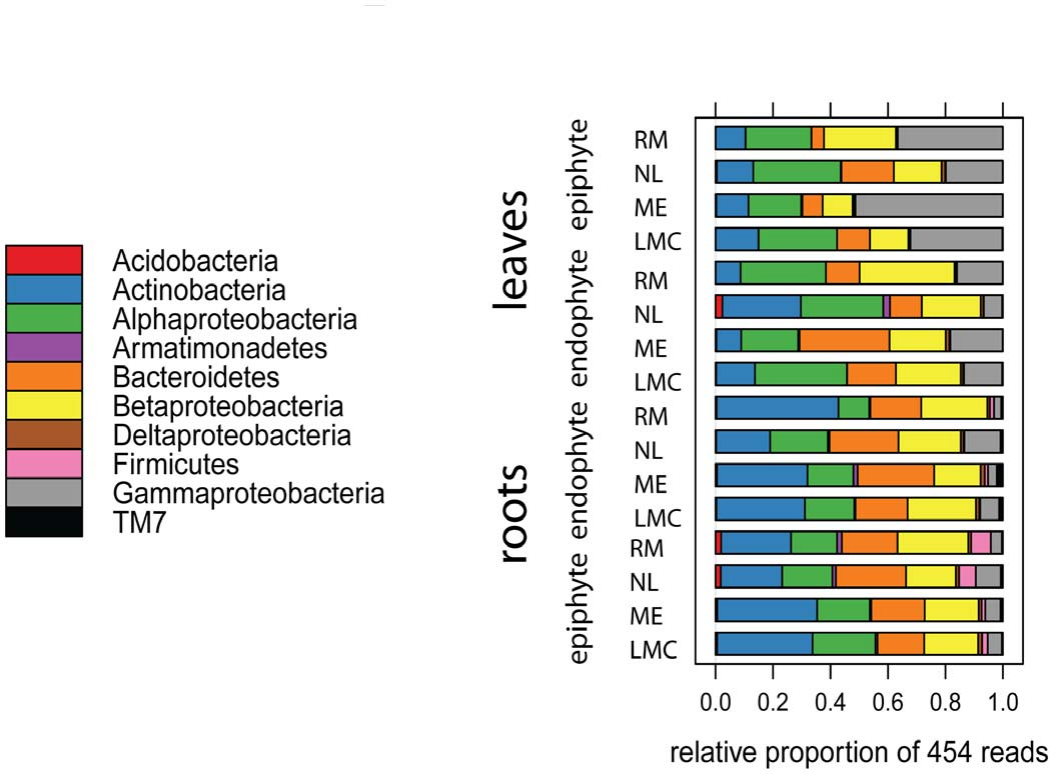
Relative abundance of bacterial phyla associated with *Arabidopsis thaliana* roots and leaves. The Proteobacteria OTU has been replaced by 4 OTUs at the subclass level (alpha, beta, gamma, delta). Only OTUs with at least 100 sequences are represented.

To compare samples, the number of sequences per sample was standardized to the minimum number of sequences in a single sample (4329 sequences). First, taxonomy of the sequences was examined at the phylum level on the basis of the RDP Bayesian classifier. The most heavily-sequenced phyla associated with both roots and leaves were *Proteobacteria*, *Actinobacteria*, and *Bacteroidetes*; sequences assigned to the *Firmicutes* were additionally present in the rhizosphere (Figure 2). Sequences assigned to *Actinobacteria* were more abundant in the root-associated communities (28.4% of the epiphytic, 30.9% of the endophytic community) compared to the leaf-associated communities (12.3% of the epiphytic, 14.5% of the endophytic community), while sequences assigned to the class *Gammaproteobacteria* were more abundant in the leaf epiphytic samples (34.9%) compared to the leaf endophytic community (13.5%) and root-associated communities (5.7% of the epiphytic, 6.2% of the endophytic community).

We notice that predominance at the phylum and class level is driven by the high abundance of one or two OTUs (Table 1). For example, *Gammaproteobacteria* were dominant in the leaf epiphytic community due to the large number of sequences of *Pseudomonas;* similarly, in the roots, *Actinobacteria* were mostly represented by the OTUs *Actinomycetales* and *Actinoplanes*. The phylum *Bacteroidetes* is mainly represented by sequences belonging to 3 OTUs: *Flavobacterium, Chitinophagaceae* and, *Flavobacteriaceae.*

**Table 1:** The core community associated with roots and leaves of *Arabidopsis thaliana.*

OTU number	Genus (or higher)	Phylum	Leaf-associated				Root-associated				Result of ANOVA test					
			endophyte		epiphyte		endophyte		epiphyte		organ		habitat		interaction	
			average propotion		average propotion		average propotion		average propotion		F	*P*	F	*P*	F	*P*
Otu006	Flavobacteriaceae	Bacteroidetes	2.56%		1.77%		2.25%		2.60%		0.21	0.657	0.80	0.389	0.87	0.370
Otu007	Methylobacterium	Proteobacteria	3.24%	a	2.56%	a	0.91%	b	0.62%	b	35.11	6.97E−05	1.33	0.270	0.00	0.975
Otu008	Otu008	Proteobacteria	3.10%		2.37%		1.99%		2.10%		3.18	0.100	0.56	0.468	1.45	0.252
Otu009	Otu009	Otu009	3.55%		6.77%		7.09%		9.32%		6.26	0.028	2.20	0.164	0.15	0.710
Otu023	Pseudomonas	Proteobacteria	10.90%	b	31.40%	a	2.02%	c	2.31%	c	56.67	6.97E−06	6.28	0.0276	3.21	0.0986
Otu027	Actinomycetales	Actinobacteria	2.67%	ab	0.86%	a	2.06%	ab	3.41%	b	5.50	0.037	0.45	0.514	9.53	0.00942
Otu030	Oxalobacteraceae	Proteobacteria	4.16%		4.13%		4.53%		3.73%		0.02	0.901	0.24	0.634	0.09	0.769
Otu031	Chitinophagaceae	Bacteroidetes	0.95%	ab	0.86%	a	3.54%	bc	4.90%	c	23.07	4.31E−04	0.16	0.696	0.42	0.530
Otu034	Sphingomonas	Proteobacteria	8.26%	a	9.16%	a	1.90%	b	2.04%	b	100.39	3.51E−07	0.70	0.418	0.06	0.816
Otu042	Burkholderiales	Proteobacteria	1.81%	a	0.63%	b	1.80%	a	2.43%	a	10.19	0.00775	2.49	0.141	9.98	0.00824
Otu045	Massilia	Proteobacteria	3.09%		3.39%		5.60%		4.32%		1.43	0.256	0.28	0.608	0.01	0.942
Otu047	Flavobacterium	Bacteroidetes	9.77%		3.93%		10.15%		5.89%		1.03	0.331	7.94	0.0155	0.31	0.590
Otu051	Otu051	Proteobacteria	1.25%		1.84%		2.47%		1.68%		2.10	0.173	0.10	0.753	1.69	0.218
Otu055	Microbacteriaceae	Actinobacteria	1.15%		2.67%		1.65%		1.68%		0.66	0.434	1.62	0.228	0.50	0.493
Otu059	Comamonadaceae	Proteobacteria	2.69%		2.40%		1.90%		1.80%		1.71	0.215	0.03	0.869	0.03	0.873
Otu061	Actinoplanes	Actinobacteria	1.21%	ab	0.25%	b	3.58%	a	1.05%	ab	6.13	0.0292	6.05	0.0301	0.52	0.485
Otu088	Rhizobium	Proteobacteria	2.14%		2.78%		1.08%		0.94%		4.20	0.0630	0.14	0.716	0.56	0.467
Otu090	Arthrobacter	Actinobacteria	0.29%	a	0.31%	a	0.97%	ab	2.44%	b	10.22	0.0077	1.89	0.194	1.79	0.205
Otu119	Variovorax	Proteobacteria	3.19%	ns	2.06%	ns	1.36%	ns	1.32%	ns	8.61	0.0125	0.79	0.391	1.04	0.329
Otu142	Kineosporia	Actinobacteria	0.06%	a	0.14%	ab	5.91%	b	0.91%	ab	8.54	0.0128	1.23	0.289	1.74	0.212
Otu151	Duganella	Proteobacteria	2.73%		0.05%		0.12%		0.02%		1.38	0.263	1.81	0.203	1.13	0.309

Letters show result from Tukey's ‘Honest Significant Difference’ test.

We define the core community as the 10 most abundant OTUs of each of the 4 habitats (root endophytes, root epiphytes, rosette endophytes, rosette epiphytes), resulting in 21 OTUs altogether (Table 1); these OTUs constitute 67% of the total sequences. ANOVA was used to test the effect of ‘habitat’ (epiphyte vs. endophyte) and ‘organ’ (root vs. leaf) on the relative abundance of the members of the core community. The Tukey's ‘Honest Significant Difference’ method was performed to compare average proportions. Relative abundances of 3 OTUs were found to be higher in the leaves: *Pseudomonas, Sphingomonas* and *Methlybacterium*. Furthermore, *Pseudomonas* was found to be more abundant in the leaf epiphytic community compared to the leaf endophytic community. Relative abundance of one OTU, *Chitinophagaceae,* was found to be higher in the roots. In addition, relative abundance of *Burkholderiales* was lower in the leaf epiphytic community compared to the other three communities, *Arthrobacter* was higher on the root surface compared to the leaf-associated communities and the relative abundance of *Kineosporia* was higher in the inside of the root compared to the leaf endophytic community. The other OTUs in the core community were generalist OTUs, for example *Flavobacterium* and *Massilia*. In addition, 2 OTUs that could not be classified below the phylum level, OTU8 and OTU9, were very ubiquitous genera, comprising more than 2% sequences in each sample.

We compared ranks of the most heavily-sequenced genera in the leaf and root associated communities (Figure S1). We used Kendall’s coefficient of concordance [27] to test for independence of rankings of the genera in each habitat. The top 50 genera in the leaf communities are concordant (Kendall's W  =  0.0848, Friedman's chi-square  =  4.54, *P* = 7.56e−5, 999 permutations) but not the top 50 genera within the root communities, indicating that OTUs in the phyllosphere are found together.

**Figure 3:**
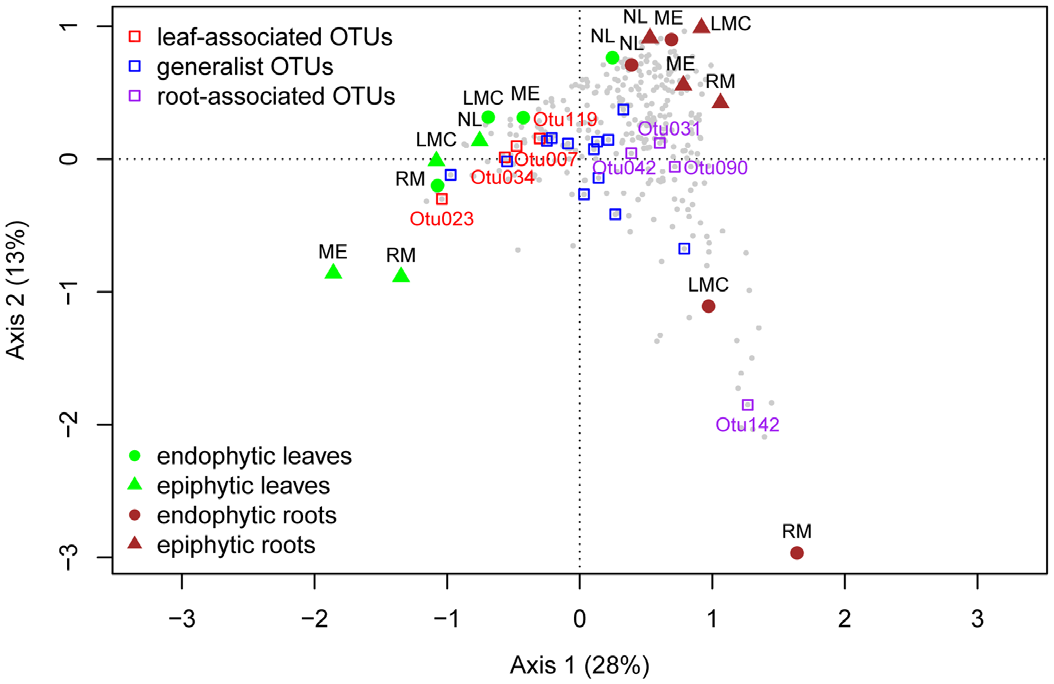
Correspondence analysis illustrates differences between bacterial communities in roots and leaves at 4 sites. The two axes represent 41% of the inertia. OTUs are represented in gray; OTUs from the core community are highlighted in three colors: red OTUs are more abundant in the leaf-associated community while purple OTUs are more abundant in the root-associated community, generalist OTUs are labeled in blue.

We performed correspondence analysis to analyze whether certain species occur at certain sites. The first axis separates samples based on organ type while the second axis separates samples based on site (Figure 3). Most samples cluster closely together, indicating that the communities are similar; however, communities in the inside of the roots of LMC and RM are relatively distinct from others, and this is correlated with more sequences assigned to *Kinesporia*. Similarly, leaf epiphytic communities of ME and RM were relatively distinct and this was correlated with more sequences assigned to *Pseudomonas*.

### Organ and Habitat Type Differentiate Communities

**Figure 4:**
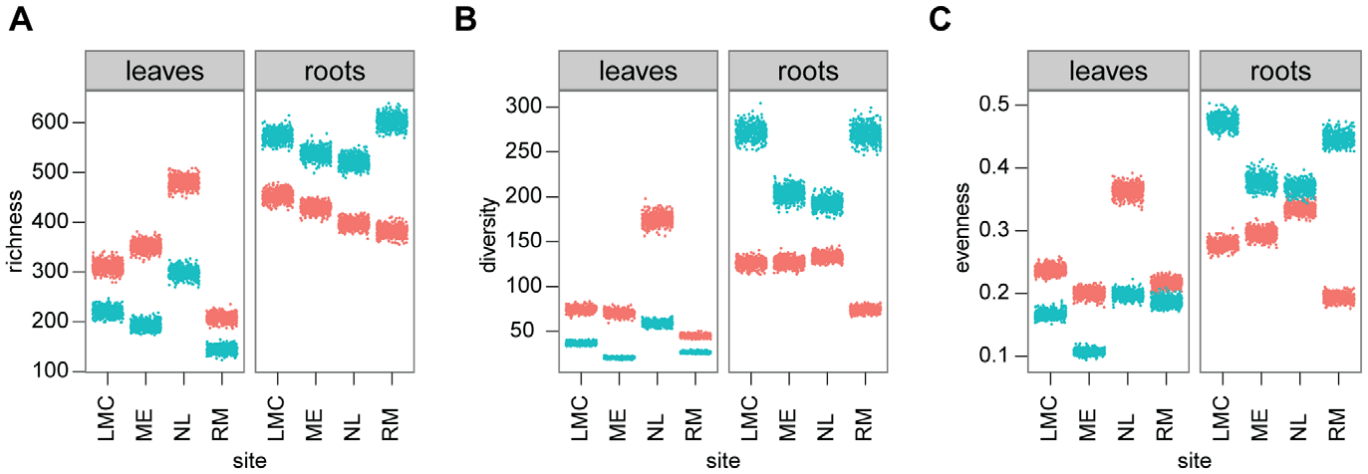
Alpha diversity of the bacterial communities in the leaves and roots of *Arabidopsis thaliana*. (A) Richness; (B) Diversity; (C) Evenness. Color represents habitat (blue, epiphytic community; red, endophytic community). Dots show the distribution of results of 500 subsampling (2000 sequences subsampled from each sample).

One of our goals was to compare bacterial communities associated with leaf and root. First, we examined differences in alpha diversity, which measures the diversity within each sample [35], focusing in particular on richness, diversity and evenness. Singletons, OTUs with only 1 sequence, were removed before calculating these indices, because singletons could be due to sequencing artifacts. 3514 singletons were removed, leaving 3160 OTUs (126841 sequences). We observed that the percent of the sequences belonging to singletons is higher for the root-associated community than for the leaf-associated community (3.22% versus 1.32% respectively; paired *t-test*, *t* = −3.8967, *P* = 0.00592). Tables of OTUs at 97% identity were subsampled 500 times for each sample and diversity indices were calculated for each permutation (Figure 4). We compared the diversity indices using pairwise *t-tests* (Figure S2). For this analysis, the average of the 500 subsamples was considered. Paired *t-tests* showed that richness is lower in the leaf epiphytic samples compared to the leaf endophytic samples (*P = *0.032) and lower compared to both root communities (*P* = 0.024); by contrast, richness is higher in the root epiphytic samples compared to the root endophytic samples (*P* = 0.024). For diversity, paired *t-tests* showed that both root communities are more diverse than the leaf epiphytic communities (*P* = 0.024 for both tests). For evenness, paired *t-tests* showed that evenness is lower in leaf epiphytic communities compared to the root epiphytic community (*P* = 0.019).

Next, we compared beta diversity [35],which is the variation in species composition. We tested the effect of ‘organ’ (leaf vs root) and ‘habitat’ (epiphyte vs. endophyte) on beta diversity. The *adonis* test found a significant effect of ‘organ’ (*F*_1,12_ = 5.64, *P* = 0.001), with pairwise dissimilarities between root and leaf samples higher than within root or within leaf samples. Similarly, the *mrpp* and *anosim* tests also found a significant effect of organ (both tests: *P* = 0.001) but no significant effect of site or habitat.

## Discussion

The most heavily sequenced members of the bacterial communities associated with *Arabidopsis thaliana* roots and leaves were related to described species; 60% of our sequences could be assigned at the genus level. Our ability to assign sequences at the genus level is lower than results obtained in a pyrosequencing study of the potato rhizosphere (75%) [36] but higher than a pyrosequencing study of the spinach phyllosphere (54%) [37]. In general, our study – and these other pyrosequencing studies - find lower representation of culturable species than clone library studies, which have found that 85 to 95% of sequences can be attributed to known genera [20], [38], [39]. This difference is due to the much higher sequencing depth of pyrosequencing. Of course, both clone libraries and 16S rRNA pyrosequencing underestimate true diversity due to primer bias. The primer used in this study (799f), for example, was designed to avoid amplification of chloroplast and therefore excludes Cyanobacteria. In fact, primer 799f matches only about 62% of the sequences in the RDP database (using Probe Match tool on RDPII).

*Proteobacteria*, *Actinobacteria*, and *Bacteroidetes* were the most abundant phyla associated with *A. thaliana*, all phyla that are typical of the phyllosphere [20], [40] and the rhizosphere [21], [22], [39], [41], suggesting substantial overlap in the key community members across host species. That said, there are many bacterial groups common on other hosts that we did not observe on *A. thaliana.* For example, the tree phyllosphere is heavily populated by *Deinococcus-Thermus* and TM7 [40]; all of these are rare in our samples. Moreover, we did not observe many sequences for the *Enterobactericeae*, which dominate the spinach phyllosphere [37] or for *Bacillus* and *Pantoea,* which dominate the lettuce phyllosphere [42], nor did we find any *Rheinheimera* sequences (and very few sequences for the genera *Dyadobacter*, *Devosia* and *Pedobacter*), which are abundant in the potato root communities [36]. On the other hand, *Rathayibacter*, found in the *Arabidopsis* phyllosphere, is not present in spinach, lettuce, or potato.

Comparison of the communities associated with the leaves and roots reveals both ubiquitous and organ specific groups. *Flavobacterium* (from 4% to 10% sequences) and *Sphingomonas* (from 2 to 9% sequences) are two abundant genera in both root and leaf associated communities which have potentially beneficial effects for plant growth and health. The abundance of *Flavobacterium,* a common soil and water bacterium, was positively correlated with potato biomass [36]. *Sphingomonas* has been isolated from a variety of environmental and plant samples [43] and some strains have a protective effect against plant pathogens [17]. *Pseudomonas* sequences are common in all four sample types, but they are most abundant in the leaf epiphytic community (31% sequences). In addition, sequences of *Methylobacterium*, a common phyllosphere colonizer [20], were also found in root samples (0.6 to 0.9% sequences). All these groups were previously known to be abundant on *A. thaliana* as based on culture-dependent surveys [44], culture-independent studies [36] and proteomic screens [20]. Overall, leaves and roots of *A. thaliana* are colonized by many of the same genera, albeit in different proportions. This suggests that many of the taxa found in the leaves and roots of *A. thaliana* may come from similar sources. Since *Arabidopsis* leaves are close to the ground, bacteria in the leaves may come from rain splashing off the soil. In fact, some soil particles could be observed on the leaves at the time of sampling. Conversely wind and rain, thought to be a source of bacteria in the phyllosphere, also bring bacteria to the soil. A third explanation is that seeds are colonized from the soil, and as the plant grows, bacteria colonize the expanding leaves.

An important caveat applies to interpretation of community studies that utilize pyrosequencing. The genus with the highest number of sequences is not necessarily the most abundant in the community due to several factors, including primer bias and 16S rRNA operon copy numbers. 16S rRNA copies range from 1 to 15 depending on the bacterial species [45]. The proportion of *Sphingomonas,* which has two copies of 16S rRNA, would thus be underestimated relative to *Pseudomonas,* which has five copies (rrnDB, Lee et al. 2009). Estimation of community composition based on 16S libraries should ideally take into account the copy number [46]; however, this is not yet feasible with 454 pyrosequencing, because it would be necessary to assign sequences to the species level and the number of species in the database is low (as of Oct 4 2012, 1411 species in the rrnDB).

Environmental variability promotes diversity [13], [14], and for this reason we expected the phyllosphere, generally thought to be quite variable, to be more diverse than the rhizosphere. However, we found the opposite: the root epiphytic community was richer and more diverse than leaf epiphytic community. There are several reasons why this might be true, and we cannot distinguish them. First, the soil environment is actually heterogeneous at the micrometer scale; it is made up of different components (e.g. sand, silt, clay, organic matter) with different chemical properties which create very different microhabitat along the root [47]. Moreover, the rhizosphere is quite dynamic and complex because of the large and diverse amount of secreted plant exudates [48]. Third, the soil harbors a very diverse bacterial community, which may be a source of endophytic colonizers [49]. In addition, we found differences in the beta-diversity of the leaf and root associated communities, which reveal differences in the composition of microbes associated with these plant organs. Similar differences in the composition of root and shoot associated bacterial communities have been found on potato [15]. These differences in composition might be due to the fact that root and leaf tissues carry different total bacterial population sizes. The bacterial abundance in the phyllosphere is estimated to be 10^7^ cells/cm^2^ [50] or roughly 10^6^ cells/g [42]. By contrast, bacterial abundance in the rhizosphere may reach up to 10^8^ cells/g dry weight root tissue [51]. Diversity has been shown to be positively correlated with total community size [44].

Communities associated with the outside of the roots were colonized by a greater number of species than in the endophytic compartment, confirming results from a study in poplar [52] and Arabidopsis [21]. Interestingly, the reverse pattern was found in the leaves: communities associated with the outside had lower richness than the endophytic compartment. We expected higher richness in the leaf epiphytic communities based on a previous study in our lab that relied on culturing microbes on *A. thaliana* [44]. In addition, bacteria are generally thought to first colonize the leaf surface and then colonize the internal space of the leaves [53]. However, there is also evidence that bacteria from the soil first colonize the roots and then migrate to the above-ground part of the plant: for example, GFP-tagged beneficial bacteria such as *Rhizobia* inoculated in the soil were found in leaves [54]; similarly, pathogenic bacteria such as *Dickeya* were found in stems [55]. We speculate that over time, bacteria from the root endophytic compartment migrate or are transported to the leaf endophytic compartment, explaining the higher richness in that compartment compared to the leaf epiphytic community. We found several potential movers in the core community: Burkholderiales, which were quite abundant in both root habitats as well as the leaf endophytic community but significantly less abundant in the leaf epiphytic community, as well as Actinomycetales and Actinoplanes, which follow a similar pattern. Indeed, both root and leaf endophytic compartments were colonized by a similar number of OTUs, suggesting that the two compartments may form a continuum.

## Supporting Information

Figure S1 **Rank abundance of the 50 most heavily sequenced OTUs.** Roots (left) and leaves (right). OTU numbers were replaced with GENUS, FAMILY or ORDER name depending on the level at which this OTU could be assigned. *Arabidopsis thaliana* were collected at 4 sites (purple, Route Marker; blue, North Liberty; green, Michigan Extension; red, Lake Michigan College). DNA was extracted for endophytic fraction (circle) and epiphytic fraction (triangle).
(TIF)

Figure S2 **Alpha diversity of the bacterial communities in the leaves and roots of** ***Arabidopsis thaliana*** **.** (A) Richness; (B) Diversity; (C) Evenness. Bars represent one standard error of the mean. The letters indicate results from paired t-test (*P*<0.05, P values adjusted using fdr).
(TIF)
