# Supplementary figures and images for: Bacterial Communities Associated with the Leaves and the Roots of Arabidopsis thaliana
Source: PLoS One. 2013 Feb 15;8(2):e56329. doi: 10.1371/journal.pone.0056329 (PMC3574144; doi:10.1371/journal.pone.0056329)

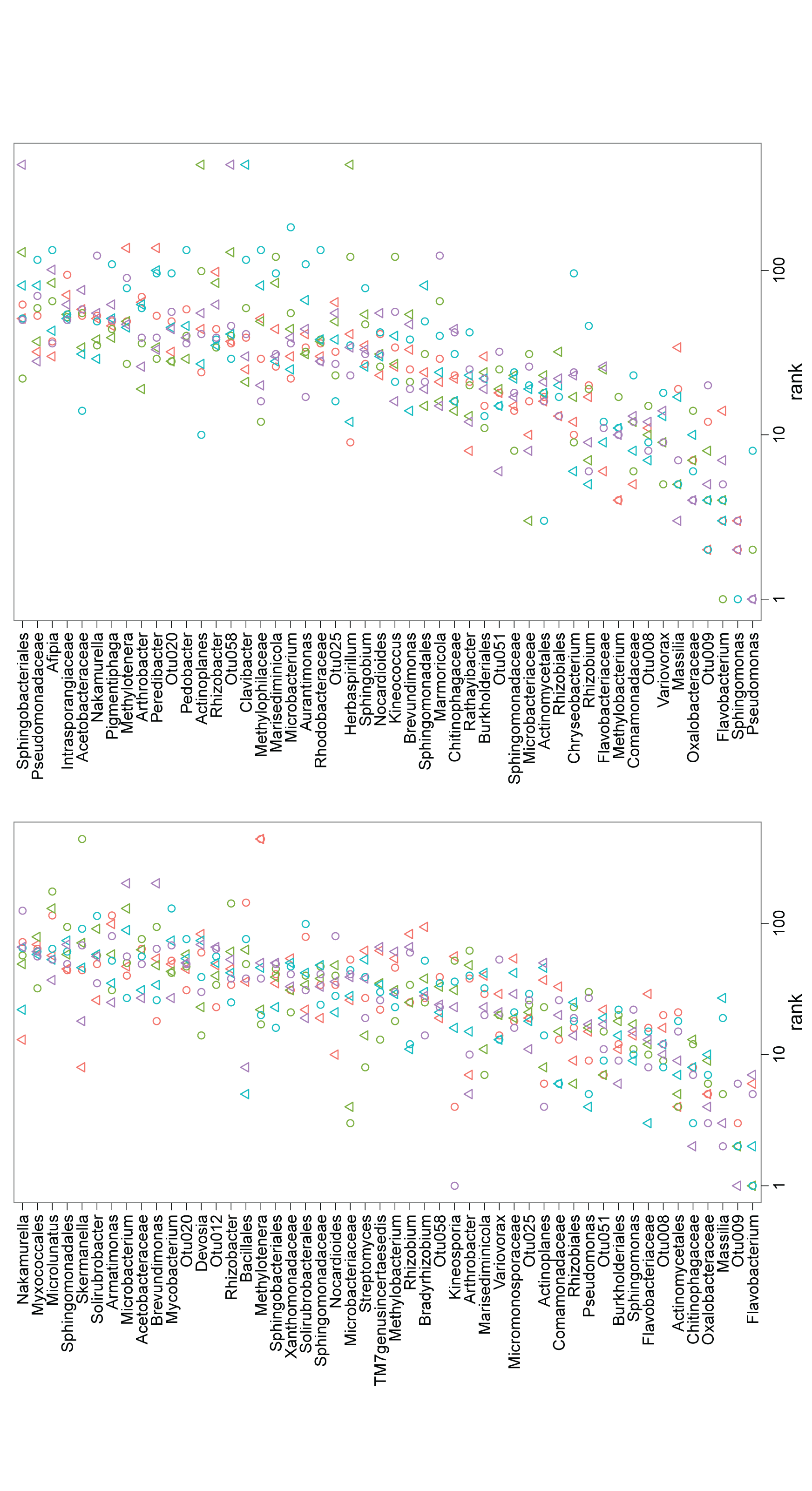

Supplement: Figure S1 — Rank abundance of the 50 most heavily sequenced OTUs. Roots (left) and leaves (right). OTU numbers were replaced with GENUS, FAMILY or ORDER name depending on the level at which this OTU could be assigned. Arabidopsis thaliana were collected at 4 sites (purple, Route Marker; blue, North Liberty; green, Michigan Extension; red, Lake Michigan College). DNA was extracted for endophytic fraction (circle) and epiphytic fraction (triangle). (TIF) [file pone.0056329.s001.tif]

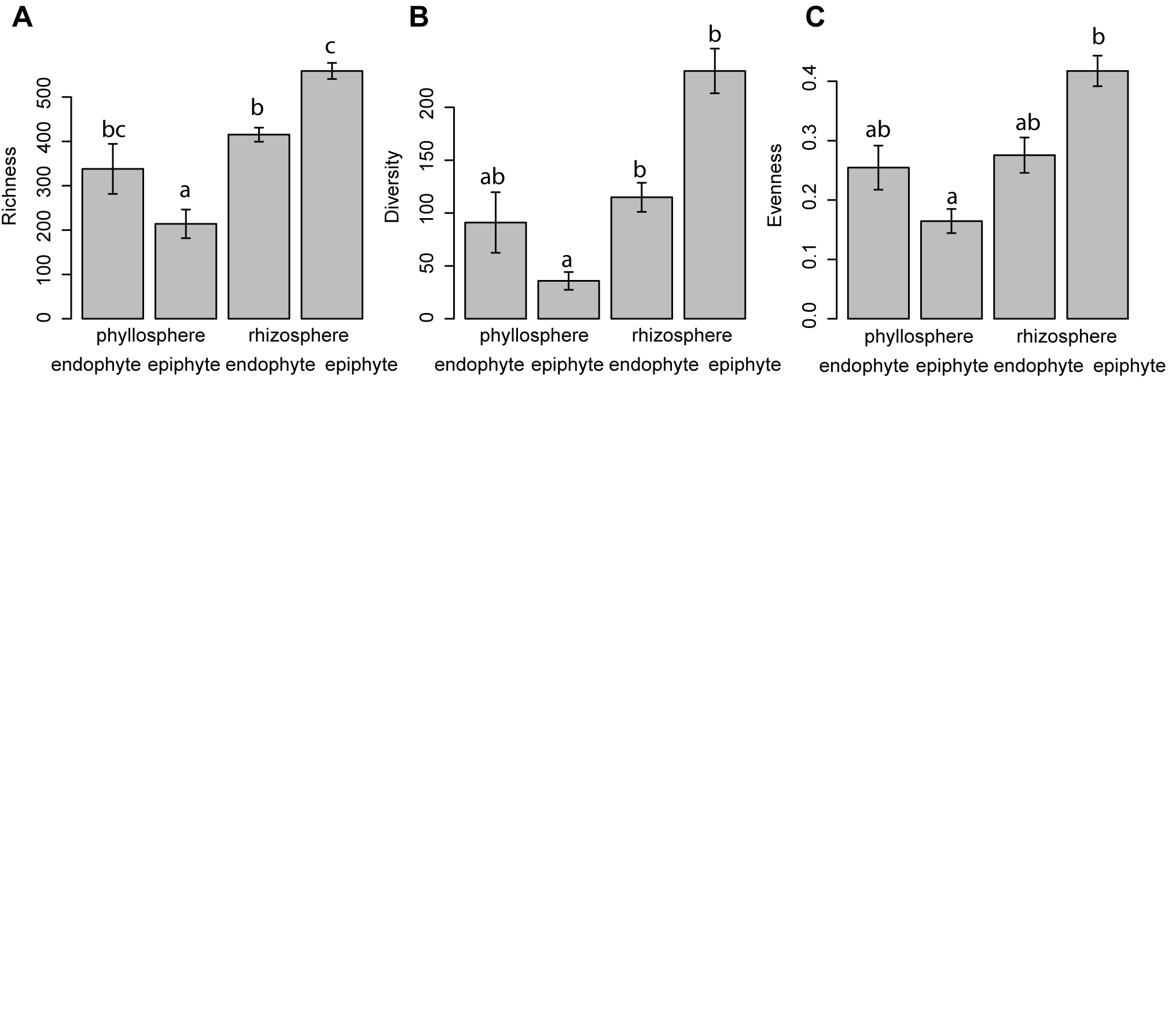

Supplement: Figure S2 — Alpha diversity of the bacterial communities in the leaves and roots of Arabidopsis thaliana . (A) Richness; (B) Diversity; (C) Evenness. Bars represent one standard error of the mean. The letters indicate results from paired t-test (P<0.05, P values adjusted using fdr). (TIF) [file pone.0056329.s002.tif]
